# Image Aesthetic Assessment Based on Image Classification and Region Segmentation

**DOI:** 10.3390/jimaging7010003

**Published:** 2020-12-27

**Authors:** Quyet-Tien Le, Patricia Ladret, Huu-Tuan Nguyen, Alice Caplier

**Affiliations:** 1 GIPSA Lab, Grenoble Alpes University, 11 rue des Mathematiques, Grenoble Campus BP 46, F-38402 Saint Martin d’Heres CEDEX, France; patricia.ladret@gipsa-lab.grenoble-inp.fr (P.L.); alice.caplier@gipsa-lab.grenoble-inp.fr (A.C.); 2 Faculty of Information Technology, Vietnam Maritime University, 484 Lach Tray, Le Chan, Hai Phong 04000, Vietnam; huu-tuan.nguyen@vimaru.edu.vn

**Keywords:** image aesthetic assessment, region of interest, sharpness map, color saliency map, large field image, close-up image, image classification, exif, handcrafted features, learned features

## Abstract

The main goal of this paper is to study Image Aesthetic Assessment (IAA) indicating images as high or low aesthetic. The main contributions concern three points. Firstly, following the idea that photos in different categories (human, flower, animal, landscape, …) are taken with different photographic rules, image aesthetic should be evaluated in a different way for each image category. Large field images and close-up images are two typical categories of images with opposite photographic rules so we want to investigate the intuition that prior Large field/Close-up Image Classification (LCIC) might improve the performance of IAA. Secondly, when a viewer looks at a photo, some regions receive more attention than other regions. Those regions are defined as Regions Of Interest (ROI) and it might be worthy to identify those regions before IAA. The question “Is it worthy to extract some ROIs before IAA?” is considered by studying Region Of Interest Extraction (ROIE) before investigating IAA based on each feature set (global image features, ROI features and background features). Based on the answers, a new IAA model is proposed. The last point is about a comparison between the efficiency of handcrafted and learned features for the purpose of IAA.

## 1. Introduction

Nowadays, the development of technology leads to the dramatic increase of digital photos since photos can be taken easily by using smartphones, tablets, laptops, cameras … Users have to confront with the lack of storage so they cannot keep all photos. Thus, there is a need of evaluating photos automatically to keep the best ones and especially to remove the worst ones. One of the most important criteria for assessing photos is image aesthetic. Beside that, image aesthetic features are the base for many applications such as image quality enhancement, photo management and sharing applications, … Therefore, studying image aesthetic could help improving several applications.

**Figure 1:**
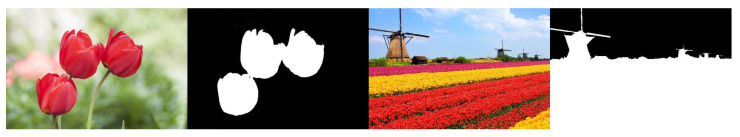
Example of close-up images (on the left), large field images (on the right) and the corresponding ROI map (the binary images).

**Figure 2:**
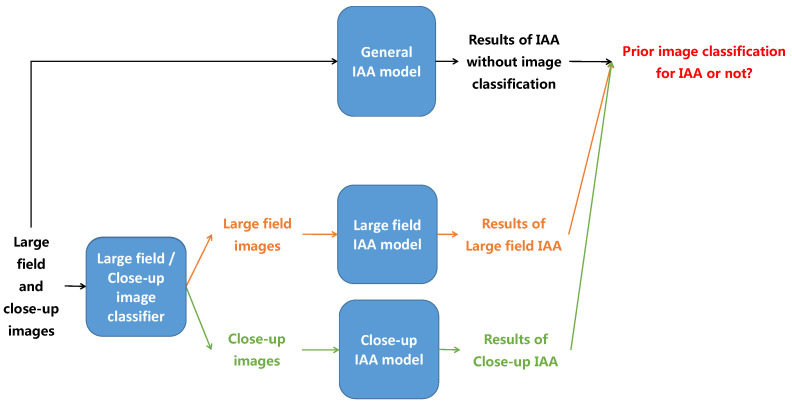
The process of image aesthetic study based on LCIC results.

Image aesthetic is an abstract notion related to the measure of delight or annoyance of an observer about a photo fulfilling aesthetically or not his/her expectations. When looking at an image, sharp and/or salient color regions often attract more viewers’ eyes while background areas often get less viewers’ attention. Thus, sharpness and color saliency are two factors defining the Region of Interest (ROI) we are looking for. In Figure 1, the first photo is a close-up image of tulip flowers while the third photo is the large field scene of a tulip field. In the close-up photo, the blur background and the high contrasted colors between the flowers and the background are exploited to highlight the sharp and high contrasted color flowers so the background is not consider as a bad quality area of the image even if it is blurry. On the contrary, although the main objects in the right photo are the colorful tulip field and the windmills, the roles of the blue sky and white clouds are significant in the aesthetic quality of the image because the whole image is considered when assessing aesthetic of large field images. The two image categories focused here are large field images (images of large field scenes taken with a long distance from the camera to the scene) and close-up images (images focusing on close-up objects captured with a short distance from the camera to the objects) because of the obvious differences of photographic rules and aesthetic evaluation criteria between them. Moreover, those both categories contain a huge amount of possible images. Based on this intuitive idea, the first contribution of this work is to demonstrate more rigorously if an image classification between large field and close-up images before IAA is worthy. The primary idea here is to assess image aesthetic of large field and close-up images separately and to consider different aesthetic features for both image categories. The illustration of the proposed process is presented in Figure 2. Images are first classified as large field or close-up images. Aesthetic quality of the two categories is then assessed separately as high or low with two different classifiers: one designed for large field images and the other designed for close-up images. Those results are compared with the results of IAA without prior classification to evaluate the influence of LCIC in IAA.

**Figure 3:**
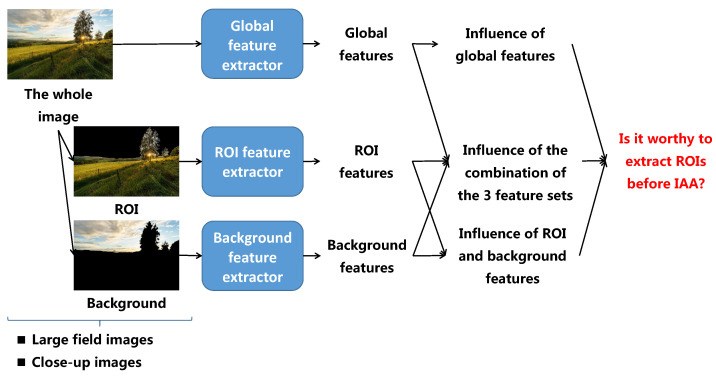
The process of image aesthetic study based on ROIE results.

Secondly, as a matter of fact, there is an implicit assumption that the aesthetic quality of an image is more related to the aesthetic quality of the ROI in this image than on the aesthetic quality of the whole image. Looking at Figure 1, the ROIs (represented by white regions) are more salient and attract more viewers’ attention than the background (represented by the black regions). The second contribution of the paper is then to investigate if it would be worthy to extract some ROIs before IAA. The illustration of the idea is presented in Figure 3. Looking at the process, the first step is to extract the ROIs and the background from an input image. Aesthetic features are then computed from the whole image, the ROIs and the background. IAA based on each feature set (global image features, local features including ROI features and background features) are performed and compared with IAA based on both global and local features to evaluate the roles of ROIE in IAA. This problem is studied in two cases: IAA for large field images only and IAA for close-up images only because large field images and close-up images are two typical image categories having opposite photographic rules related to ROIs and background.

The third contribution of the paper is to compare the efficiency of handcrafted features and learned features for the purpose of IAA. Aesthetic features are computed either by hand or via a learning algorithm.

Based on the evaluations of LCIC and ROIE in IAA, a new IAA model is finally proposed.

There are two additional contributions regarding pre-processing for IAA. An ROIE algorithm using the combination of sharpness and color contrast information and a deep model are introduced. The second contribution is to consider different types of features including Exchangeable Image File Format (EXIF) features, handcrafted features and learned features to perform the Large field/Close-up Image Classification (LCIC) task.

This paper is organized as follows. Section 2 presents a state of the art about IAA, ROIE and LCIC. Section 3 describes the proposed pre-processings for IAA: on one side, ROIE based on both sharpness, color information and on the other side, LCIC based on EXIF features, handcrafted features and learned features. Section 4 is to define features for IAA. The study of IAA with prior image classification is described in Section 5. Section 6 presents the study of IAA with prior region segmentation. The conclusions and a new IAA model based on LCIC and ROIE are drawn in the last section.

## 2. State of the Art

### 2.1. Image Aesthetic Assessment Studies

Many attempts have been made to train computers how to automatically assess the aesthetic quality of images. Generally, there are two main phases in an IAA process [1]. The first one is to extract features from images: handcrafted features or learned features. In the second phase, a decision is made. The decision could be a binary classification indicating the input image as high or low aesthetic. It also could be a regression decision (returning aesthetic scores) or aesthetic ranking orders.

Following handcrafted approaches, most of studies focus on photographic rules to design aesthetic features. Dhar et al. [2] propose to use low level features to form high level features for IAA. There are three groups of features including compositional features (presence of a salient object, rules of composition, depth of field, opposing colors), content features (presence of objects or object categories) and Sky-Illumination features (natural illumination). A Support Vector Machine(SVM) classifier is trained to predict aesthetic and interestingness by using 26 high level features. In [3], an IAA method using a generic content-based local image signature is proposed. Bag of visual words descriptors, Fisher vector and GIST descriptors are considered to form generic content-based features. Bag of visual words descriptors, Fisher vector, gradient information are encoded by using SIFT and color information. Two SVM classifiers are trained for binary image aesthetic classification, one with SIFT and the other with color features. The average of the two results is considered as the final result. Mavridaki et al. [4] propose to use five feature groups including simplicity, colorfulness, sharpness, pattern and composition to perform IAA. Their feature vector is constructed from both low and high level features computed on both the whole image and local regions. In [5], Aydin et al. introduce an aesthetic signature concept and an aesthetic quality assessment method based on sharpness, depth, clarity, tone and colorfulness features. Their results prove that the aesthetic signature can help improving automatic aesthetic judgment, automated aesthetic analysis, tone mapping evaluation, …

Deep learning approach might be a good solution for IAA and many researches about image aesthetic using deep learning have been introduced. Tian et al. [6] introduce a query-dependent aesthetic model based on deep learning for IAA. They combine a retrieval system and a deep Convolutional Neural Network (CNN) to improve the performance of IAA. Given an input image, visual features and textual features are extracted first as the input for the retrieval system. Images in similar categories are retrieved to construct a training set for the aesthetic model. The model is then trained on the constructed training set to predict aesthetic labels. Their idea is interesting but the execution time could be an issue since whenever evaluating the aesthetic quality of an image, a retrieval task has to be executed first and the aesthetic model then has to be trained before predicting aesthetic labels. In [7], a double-column deep CNN is proposed to perform IAA. Two parallel CNNs are used: one learning aesthetic features from the whole image and the other learning aesthetic features from local parts. Those features are then combined to classify images as high or low aesthetic quality. Additionally, style and semantic attributes are leveraged in their work. In [8], Wang et al. introduce an CNN including three groups of layers to evaluate image aesthetic of multi-scenes. The first group of layers contains four convolutional layers pre-trained on the ImageNet dataset. The second one consists of seven parallel groups in which each group is corresponding to a kind of scene in the CUHKPQ dataset (animal, architecture, human, landscape, night, plant and static). Each group of layers is pre-trained on the corresponding image group of the CUHKPQ dataset. The last group includes three fully connected layers to evaluate image aesthetic as high or low. Their model is a combination of transferred layers, scene convolutional layers and fully connected layers.

In general, image aesthetic has been studied in various ways in which prior region segmentation [5,9,10] or prior image classification [6] have been considered. However, those studies focus mainly on applying prior region segmentation and prior image classification in IAA (how to exploit or apply them in IAA? How good the performances of methods are?). On the contrary, our purpose is to compare the performances of IAA when considering the image dataset without any pre-processing with those obtained with pre-processing like prior image classification and/or prior image segmentation. Additionally, the question “What is the efficiency of handcrafted features with regard to learned features in IAA?” still needs to be answered. In this study, we are going to tackle those problems and a binary IAA is chosen because of its simplicity. Obtained conclusions can be extended to regression IAA.

### 2.2. Large Field/Close-up Image Classification Studies

Image classification has been studied for many years and the main idea is to use image features that are computed from image data either by hand [11,12] or via a learning algorithm [13,14] to separate images into different categories. The focused problem in this part is to classify large field images and close-up images (image samples can be seen in Figure 1). Until now, there are few researches about this particular classification. In [15], Wang et al. propose a method using color coherence vector and color moments to classify close-up and non close-up images. In another study, Zhuang et al. [16] divide an image into 256 parts. The number of edge points in each part is counted to build a 256 bin histogram. The 256 bin values and standard deviation of those values are the key features to classify close-up and distance view images. In [12], Tong et al. use features representing the distributions of high frequencies in the first classification stage. In the second one, the spatial size and the conceptual size (object size in reality) are used to classify distance/close-up view images.

All features used in those classification methods are handcrafted features. The role of EXIF features and learned features for LCIC is still an open question. Handcrafted features and learned features have been widely used for general image classification [17]. Nowadays, deep learning approaches are the must for object classification [18]. At the same time, EXIF data has not been widely used for image classification. EXIF data are metadata (data information of data) and tags revealing photo information such as picture-taking time, picture-taking conditions [19]. Surprisingly, EXIF features have been occasionally used in researches. In [20], Huang et al. use the manufacturer, camera model, date and time stamp and some other EXIF parameters as watermark information to protect image copyright. In [21], aperture, exposure value, ISO and picture-taking time are exploited to enhance ROI detection. In [22,23], Boutell et al. integrate image content and EXIF data consisting of exposure time, flash use and focal length to classify in-door and out-door images.

In this paper, the performances of LCIC pre-processing based on EXIF features, handcrafted features and learned features are compared in terms of accuracy and computational complexity.

### 2.3. Region of Interest Studies

There are many ways to extract ROIs. The first way is to consider image sharpness because viewers are often attracted by sharp and clear regions. Following this idea, from an input image, Luo et al. [24] use blurring kernels, horizontal and vertical derivatives to compute sharpness information. Each pixel is labelled as blur or clear and the ROIs are considered as the rectangular regions with the highest sharpness values. However, it is obvious that the shape of any ROI is not always rectangular. Re-using Luo’s sharpness calculation, Tang et al. [25] propose first to segment the input image into super-pixels (groups of neighboring pixels having similar colors) [26] and then the labels of neighboring pixels are used to improve the precision of ROIE. A super-pixel is determined as belonging to an ROI if over half of its pixels are labelled as clear. In [5], Aydin et al. use an edge stopping pyramid to blur the input image multiple times. By considering the differences between the blurred versions of the sequential pyramid levels, a sharpness map is computed first and the in-focus regions are then extracted based on it.

The second approach is based on the fact that regions with salient and/or high contrasted colors often get more viewers’ attention. In [27], Perazzi et al. use color contrast and color distribution to estimate the color saliency level of each super-pixel. Color variations, spatial frequencies, structure and distribution of image segments are considered in their study. In [28] an algorithm using the combination of color dissimilarity with background prior for color saliency level computation is proposed. In [29], exploiting both weak and strong models, a salient object detection method combining color saliency and bootstrap learning to extract salient regions is proposed. A weak saliency map is constructed first based on image priors to generate training samples for a strong model. Then, the strong classifier is learned to detect salient pixels from images directly. In [30], a color saliency detection method analyzing color histogram and spatial information-enhanced region based contrast is proposed.

Beside handcrafted methods, deep learning based methods have been developed for region detection and saliency prediction [31,32,33,34]. In [32], CNNs are used to modelize saliency of objects in images by considering both global and local contexts. Saliency features are extracted from two models, one trained on the global context and the other trained on local contexts. Both feature types are then used for color saliency computation. Li et al. [31] propose to use CNNs to learn saliency features from multiscale images for visual recognition tasks. Different visual saliency maps are generated from multiscale images coming from an original one. Those maps are then combined to create the final saliency map. In [34], an end-to-end deep hierarchical network based on CNN for salient object detection is proposed. The first network learns global contrast, objectness, compactness features. Then a hierarchical recurrent CNN is used to hierarchically refine the details of saliency maps by integrating local context information. Cornia et al. [33] propose to predict viewers’ attention on image pixel by using an CNN containing three main blocks: a feature extraction CNN, a feature encoding network and a prior learning network. That model extracts deep features from different levels of the CNN and combines them to predict eye fixations over the input image.

**Figure 4:**
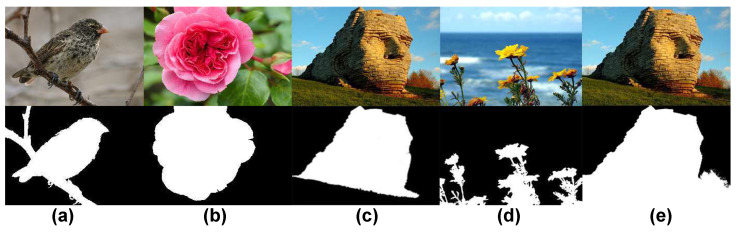
Examples of different definitions of ROIs. The first row contains color images and the second row contains the corresponding ROI maps (**a**) ROIs defined according to sharpness. (**b**) ROIs defined according to color saliency. (**c**) ROIs defined as object regions (**d**,**e**) Our ROI definition based on both sharpness AND color saliency.

In our work, ROIs are defined as regions attracting viewers’ attention because of both sharpness AND color saliency (see Figure 4d,e). They are not only sharp regions or only regions with high color saliency levels or regions containing objects (see Figure 4a, Figure 4b, Figure 4c respectively).

## 3. Pre-Processing Phases for IAA

### 3.1. Large Field/Close-up Image Classification

Before exploring the interest of a prior image classification for IAA, the aim of this section is to determine which are the best features to consider in order to proceed to LCIC.

#### 3.1.1. EXIF Features for LCIC

In photography, camera tunnings are stored by digital cameras as EXIF data. Four EXIF parameters and a combination of some of them are considered in this study.

Aperture refers to the size of lens opening for light when a picture is captured. This parameter is stored as a *f*-stops value such as f/1.4, f/2, f/2.8,…in which *f*-$stops=\frac{f}{D}$ where *f* is the focal length and *D* is the diameter of the entrance in a camera. A smaller *f*-stops value represents a wider aperture. The Depth Of Field (DOF) and brightness of pictures are affected by the setting of aperture. A decrease of the aperture value makes an increase of DOF and a decrease of brightness.

Focal length exhibits the distance from the middle of the lens to the digital sensor and it also decides the angle of view in the photo. This parameter is measured in millimeters. A long focal length makes a narrow view and a wide scene is captured with a short focal length.

Exposure time represents the total time for light falling on the sensor of a camera during shooting. It is measured in seconds. In weak light conditions, photographers use long exposure time. A short exposure time is regularly used when capturing moving objects like taking sport photos.

ISO describes the sensitivity level of the sensor in a camera. ISO parameter is measured with numbers such as 100, 200, 400, …The lower ISO value represents the less sensitive mode of the sensor. The brightness of a photo decreases with the decrease of ISO. However using a too sensitive mode could generate some noise in the taken photo.

Illumination measure refers to the light falling on a surface [35]. This feature is calculated as:

(1) $$I_{m}=\log_{10}\left(\frac{aperture^{2}}{exposure\;time}\right)+\log_{10}\left(\frac{250}{ISO}\right)$$

Considering EXIF values of 400 large field and 400 close-up photos (the training set in the next LCIC experiments) coming from the Flickr.com website, it appears that the differences of EXIF parameters between close-up and large field images are significant in aperture, focal length, illumination measure and to a smaller extent in exposure time. On the contrary, ISO feature is not relevant enough [36].

#### 3.1.2. Handcrafted Features for LCIC

**Figure 5:**
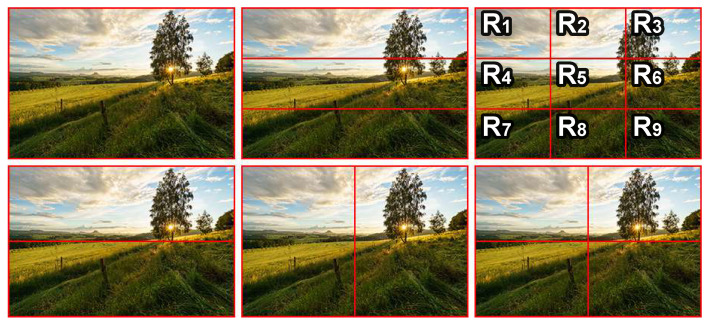
Illustrations of region splits. First row: whole scene, regions split by landscape rule and rule of thirds respectively. Second row: regions split by symmetry rules.

The main goal of this part is to build a handcrafted feature set for LCIC based on usual features computed from image data. Firstly, a large handcrafted feature set is built from common handcrafted features appearing in different researches [5,24,37,38,39]. The initial handcrafted feature set includes 2030 features related to hue, saturation, brightness, red, green and blue channels, sharpness, color saliency and contrast. Those features are global features (features computed from the whole image) and local features (features computed for different local regions). The local features are computed from ROIs, background and regions split by symmetry rules, landscape rule, rule of thirds (see Figure 5). At the next step, the feature reduction algorithm introduced in [36,40] is applied on 1200 large field images and 1200 close-up images coming from the CUHKPQ dataset [25] in which a half of them is used in the training phase ($S_{1}$) and the remaining is used in the testing phase ($S_{2}$). After the most relevant features are selected, those features are analyzed to remove overlapping features and to optimize the feature set. Twenty one features are finally considered as the most relevant for the LCIC task. (see overview of the features in Table 1).

#### 3.1.3. Learned Features for LCIC

Beside being handcrafted from images, features can also be learned by employing deep learning [41]. VGG16 [42] is a well-known deep CNN. It includes three main parts: convolutional layers, fully connected layers and a prediction layer. If the prediction layer is removed, that model can be considered as a feature extractor. From images of size 244 × 244, 4096 features are extracted by VGG16 without the last layer. Although those features have been learned for the task of classifying objects in images, they can be applied for different tasks [43] such as image quality assessment [44,45]. In this study, VGG16 without the prediction layer pre-trained on the ImageNet dataset for the task of classifying objects in images is considered to compute the learned features for LCIC on the corresponding dataset. Instead of transferring all learned features, the most relevant features are selected because some of them are pre-learned for a different task so they could not be relevant for the LCIC task.

The feature reduction algorithm described in [36,40] is run on 1200 large field images and 1200 close-up images coming from the CUHKPQ dataset to select the 925 most relevant features (the highest classification performance is obtained with those features) among the 4096 features learned by the VGG16.

#### 3.1.4. Experiment and Results

##### Dataset and Setup

LCICs are performed separately with EXIF, handcrafted and learned features. In order to evaluate the influence of the different feature types fairly, the well known SVM classifier is trained and tested to evaluate the classification performances obtained with each feature set. If complex classifiers had been used, the accuracy of the classifications could be affected not only by the input features but also by the suitability between the model structure and input features. The experiments are performed on 1600 images (with EXIF data) including 800 large field and 800 close-up images collected and categorized from the Flickr.com website by the authors. Half of the large field and close-up images are selected randomly to train the classifiers while the others are used to test. Each SVM classifier is applied with C=0.5 and different kernels: Poly, Linear, RBF and Sigmoid to find the most appropriate kernel. After performing all the experiments only the best results (with a Linear kernel) are presented. The LCIC is evaluated based on Accuracy (*A*) depending on TP,TN,FP,FN (true positive, true negative, false positive and false negative expressed as a number of images), on confidence interval of accuracy and on computational costs as described in Table 2.

The experiments have been conducted on a PC equipped with an Intel Core i7-2670QM CPU 2.40 GHz and 11.9 GB memory to evaluate the feature computational time $T_{F}$ (the time for computing features from images directly) and the classification time $T_{C}$ (the time for classifying images based on computed features) and the total computational time ($T_{T}=T_{F}+T_{C}$) per image. Additionally, the computational time for learned features is often smaller if they are computed with an GPU so an GPU NVIDIA Quadro P400 is used to compute the learned features (the computational time for handcrafted, EXIF features in this experiment are not affected by the GPU).

##### Results and Discussion

Results of LCIC using EXIF features, handcrafted features and learned features are presented in Table 3. Using a very small number of simple features (only four EXIF features), the classification accuracy at 0.878±0.023 is impressive. Additionally, the feature computational time for EXIF features is very small (under 1 ms because there is only one simple EXIF feature that needs to be computed).

The handcrafted feature set is simple since it includes only 21 features but its classification rate is also impressive (0.873±0.023). In order to prove the efficiency of our handcrafted features, the classification based on those features is compared with the classifications based on other handcrafted features including Wang’s [15] and Zhuang’s [16] features. Despite of using more features, the classifications with Wang’s (105 features) and Zhuang’s (257 features) feature sets have lower accuracy at 0.774±0.023 and 0.854±0.024 respectively. Those results prove the efficiency of our handcrafted features.

Beside that, the classification with learned features has unsurprisingly the highest overall accuracy (0.989±0.007) but the number of features is also the biggest (925 features) and the feature computational time is also the longest (434 ms - without the GPU) among the studied feature sets. With the GPU, the computational time is much smaller (16 ms).

In order to compare a litle bit more the efficiency of deep learned features with EXIF or handcrafted features, the classifications using the top 4 and top 21 most relevant learned features are performed. The comparisons between the LCICs using the reduced VGG16 feature sets and the LCICs using the handcrafted features and EXIF features are presented in Table 3. It appears that the learned features are very powerful for LCIC since with the same number of features as handcrafted features (21 features) the accuracy of the classification based on the 21 most relevant learned features is higher than that of the handcrafted features (0.981±0.009 versus 0.873±0.023). Similarly, with only four learned features as EXIF features, the accuracy of the classification based on the four most relevant learned features is 0.975±0.011, a very high accuracy while the classification accuracy with EXIF features is smaller (0.878±0.023).

Last but not least, the feature computational time and classification time per image are shown in Table 3. It is clear that EXIF features are the simplest ones when only one EXIF feature (illumination measure) needs to be computed and its feature computational time is only 1 ms. In contrast, without the GPU, the feature computational time of learned features is over 14 times of the computational time of the handcrafted features (434 ms versus 30 ms). Additionally, the feature computational costs for the 21, 925 most relevant learned features or all 4096 learned features are the same because the feature extractor always computed all 4096 features. With the GPU, the computational time of the learned features decreases significantly to 16 ms (approximately 50% of the computational time of the handcrafted features). Although the time of SVM classification based on the computed features is almost the same (1 to 2 ms), the differences in the total classification time between the different feature sets are significant. It points out that the classification based on EXIF features is very fast (only 2 ms). The classification based on handcrafted features is slower (30 ms) while without the GPU, the classification with learned features is very slow (434 ms) but the accuracy is not increasing in the same proportions. However, with the GPU, the weakness of the computational time for learned features is solved.

According to the experimental results, we conclude that learned features are very powerful for that task although they are too complex to be understood and require a strong GPU to reduce the computational time. EXIF features are quite efficient for LCIC since it is possible to obtain the same and quite good classification score by using four very simple EXIF features than by using 21 complex handcrafted features. EXIF features are simple, efficient but unfortunately they are not always available.

### 3.2. Region of Interest Extraction

Before studying the interest of prior ROIE for IAA, the aim of this section is to propose a new algorithm in order to extract the most suitable ROIs for IAA. As defined in the introduction part, in this paper ROIs are region with both high sharpness AND high color saliency.

#### 3.2.1. Handcrafted ROIE Method

As mentioned in the previous part, observers pay more attention on sharp and/or contrasted color regions. That is why we propose to define an ROIE algorithm that in the first step estimates the sharpness of all regions and in the second step computes the color saliency levels of all regions. The estimated sharpness and color saliency levels are combined to form the ROI map in the last step.

##### Sharpness Map Estimation

Normally, the in-focus regions (regions focused by photographers) are sharper than the out of focus regions so sharpness information is the primary key to detect those regions. In [5,46], they point out that when blurring a photo, the neighboring pixels’ values converge to the same gray level. The gray levels of pixels in a sharp image change significantly when the image is blurred while this change is much weaker when re-blurring a blurred image. To extract in-focus regions, a sharpness estimation method based on the combination of Aydin’s clearness map [5] and multi-scale super-pixels is introduced. Aydin’s clearness map is first calculated. Then a *k*-level edge-stopping pyramid [5] is built by using the bilateral filter [47]. The first pyramid level $L_{0}$ is the image in gray scale while the higher levels are defined as:

(2) $$L_{i}=f_{b}\left(L_{i-1},s_{i}\right)$$

where $f_{b}$ is the bilateral filter. In this work, *k* is set to 10. The kernel size at the *i*th level is $s_{i}\times s_{i}$ where $s_{i}=round\left(3\times 1.1^{i}\right)\times 2+1$. The clearness map is then calculated as the sum of absolute differences between subsequent pyramid levels as:

(3) $$M^{cl}=\sum_{i=1}^{k}\left|L_{i}-L_{i-1}\right|$$

**Figure 6:**
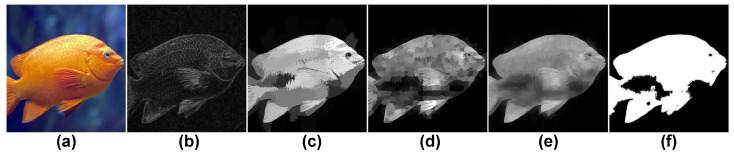
Sharpness estimation process. (**a**) original image, (**b**) Aydin’s clearness map, (**c**) sharpness distribution at level 2, (**d**) sharpness distribution at level 5, (**e**) sharpness map, (**f**) in-focus map.

Aydin’s clearness map only gives a rough estimation of the sharpness in which detected sharp pixels are located mainly on edges (see Figure 6a,b) while viewers often pay attention to the whole regions containing sharp details instead of all small sharp details. We improve the map by exploiting super-pixels. In the next step, *n* multi-scale super-pixel levels are determined. At the $i^{th}$ level, the color image is segmented into $i^{2}\times \alpha$ super-pixels (α=25, n=10 in this work). The sum of clearness values $s_{i,j}^{cl}$ of super-pixel $P_{j}$ at the *i*th level is calculated as:

(4) $$s_{i,j}^{cl}=\sum_{\left(x,y\right)\in P_{j}}M^{cl}\left(x,y\right)$$

After normalizing the $s_{i,j}^{cl}$ values to the range [0, 255], sharpness values of all pixels in each super-pixel $P_{j}$ are set to $s_{i,j}^{cl}$ and the sharpness distribution map $M_{i}^{sh}$ at the *i*th level is obtained (see Figure 6c,d for illustrations). The global sharpness map is then computed as:

(5) $$M^{sh}=\frac{1}{n}\sum_{i=1}^{n}M_{i}^{sh}$$

The sharpness map is then binarized by applying Otsu’s threshold [48] to extract the in-focus regions. The in-focus map is the binarized version of the sharpness map (see Figure 6e,f).

##### Color Saliency Map Estimation

Beside the sharpness factor, color contrast is another important factor attracting viewers’ attention. Our color saliency map is based on Liu’s idea [49] and Zheng’s idea [28] about using background and foreground priors and Perazzi’s idea [27] about using color uniqueness. Salient regions in this work are defined as regions having colors similar to the colors of the in-focus or central regions and different from the colors of out of focus regions and out of center regions (regions near photo edges) because center and in-focus regions attract more viewers’ attention than the others. Firstly, a mask is initialized based on the in-focus regions and center region:

(6) $$M^{msk}=M^{inf}\cup M^{cen}$$

where $M^{inf}$ is the in-focus map. $M^{cen}$ is a binary image in which there is a white center rectangular region of size 0.6w×0.6h while the other regions are black (*w* and *h* are the width and the height of the image). The color saliency $M_{i}^{cs}$ of super-pixel $P_{i}$ is estimated based on all out-of-mask super-pixels and all in-mask super-pixels as:

(7) $$M_{i}^{cs}=\frac{\sum_{P_{j}\in R_{oom}}d_{i,j}^{rgb}\times w_{i,j}^{p}}{\|R_{oom}\|}-\frac{\sum_{P_{j}\in R_{inm}}d_{i,j}^{rgb}\times w_{i,j}^{p}}{\|R_{inm}\|}$$

(8) $$d_{i,j}^{rgb}=\sqrt{{\left(r_{i}-r_{j}\right)}^{2}+{\left(g_{i}-g_{j}\right)}^{2}+{\left(b_{i}-b_{j}\right)}^{2}}$$

(9) $$w_{i,j}^{p}=\frac{1}{z_{i}^{p}}e^{-\frac{\sqrt{{\left(x_{i}-x_{j}\right)}^{2}+{\left(y_{i}-y_{j}\right)}^{2}}}{2\sigma_{p}}}$$

where $R_{oom}$, $R_{inm}$, $\|R_{oom}\|$, $\|R_{inm}\|$ are the out-of-mask, in-mask regions and the number of super-pixels in those regions respectively. $d_{i,j}^{rgb}$ is the color distance between the center pixels of super-pixels $P_{i}$ and $P_{j}$ in the RGB color space. Gaussian weight $w_{i,j}^{p}$ is calculated via super-pixel center positions. $x_{i}$, $y_{i}$, $r_{i}$, $g_{i}$, $b_{i}$ are the coordinates and red, green, blue intensities of the center pixel in $P_{i}$. $\sigma_{p}$ is the number of super-pixels in the image. The normalization factor $z_{i}^{p}$ ensures $\sum_{P_{j}\in R_{oof}}w_{i,j}^{p}=1$.

Pixel values in $M^{cs}$ are normalized to the range [0, 255] and the Otsu’s threshold is applied on $M^{cs}$ to create an update of the mask $M^{msk}$ and a new cycle starts. After performing this process three times, the final color saliency map $M^{cs}$ is obtained.

##### Region of Interest Map Estimation

**Figure 7:**
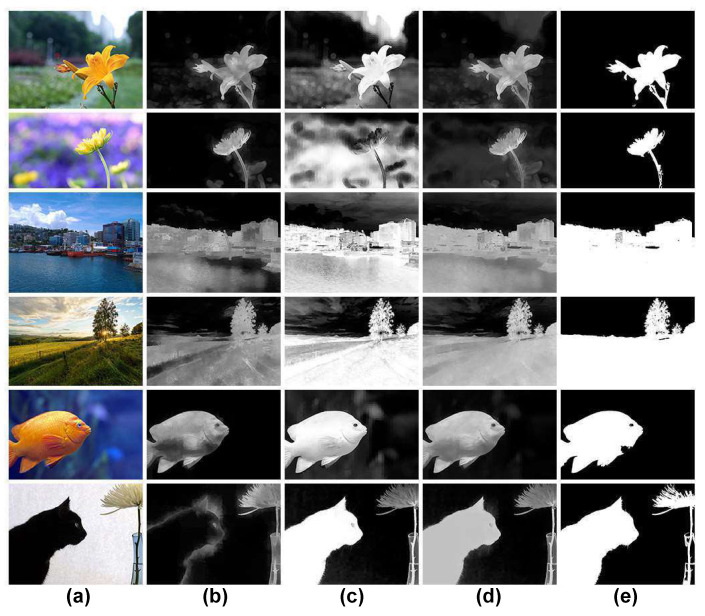
ROI map computation process. (**a**) original images, (**b**) sharpness maps, (**c**) color saliency maps, (**d**) ROI maps. (**e**) binarized ROI maps.

Looking at Figure 7, it appears that sharpness is the main factor attracting viewers’ attention in the two first rows. In contrast, the dominant criterion emphasizing the ROIs is the color saliency in the last row. For the three middle rows, both sharpness and color saliency have significant roles in highlighting the ROIs. Obviously, the influence of sharpness and color saliency factors in defining ROIs is not the same for all images. Thus, if only one of them is considered, it will not be sufficient to extract right ROIs. An algorithm combining sharpness and color saliency factors based on the spatial distribution of pixel values to extract ROIs is presented in this part. Given a gray image (a sharpness or color saliency map) *I*, the coordinates of the center point of the rectangle are first determined as:

(10) $$x_{c}=\frac{\sum_{x=1}^{w}\sum_{y=1}^{h}I(x,y)\times x}{\sum_{x=1}^{w}\sum_{y=1}^{h}I(x,y)}$$

(11) $$y_{c}=\frac{\sum_{x=1}^{w}\sum_{y=1}^{h}I(x,y)\times y}{\sum_{x=1}^{w}\sum_{y=1}^{h}I(x,y)}$$

**Figure 8:**
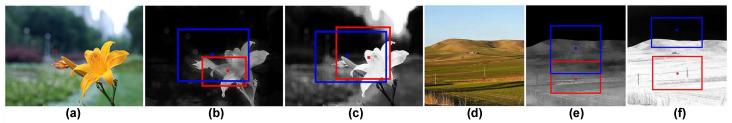
Examples of rectangles representing the distribution of pixel values. (**a**,**d**) original images, (**b**,**e**) sharpness maps, (**c**,**f**) color saliency maps. Red rectangles represent the distributions of pixel values in those images while blue rectangles reflect the distributions for the corresponding video inverted images.

These coordinates are then used to calculate the deviations as:

(12) $$d_{l}=\frac{\sum_{x=1}^{x_{c}}\sum_{y=1}^{h}I\left(x,y\right)\times |x-x_{c}|}{\sum_{x=1}^{x_{c}}\sum_{y=1}^{h}I(x,y)}$$

(13) $$d_{r}=\frac{\sum_{x=x_{c}}^{w}\sum_{y=1}^{h}I\left(x,y\right)\times |x-x_{c}|}{\sum_{x=x_{c}}^{w}\sum_{y=1}^{h}I(x,y)}$$

(14) $$d_{t}=\frac{\sum_{x=1}^{w}\sum_{y=1}^{y_{c}}I\left(x,y\right)\times |y-y_{c}|}{\sum_{x=1}^{w}\sum_{y=1}^{y_{c}}I(x,y)}$$

(15) $$d_{b}=\frac{\sum_{x=1}^{w}\sum_{y=y_{c}}^{h}I\left(x,y\right)\times |y-y_{c}|}{\sum_{x=1}^{w}\sum_{y=y_{c}}^{h}I(x,y)}$$

where $d_{t}$, $d_{r}$, $d_{b}$ and $d_{l}$ are the top, right, bottom and left deviations respectively. The rectangle $R_{I}$ representing the distribution of pixel values in the image *I* is illustrated by the red rectangles in Figure 8. The coordinates of the top left and bottom right points of $R_{I}$ are computed as:

(16) $$x_{tl}=x_{c}-d_{l}$$

(17) $$y_{tl}=y_{c}-d_{t}$$

(18) $$x_{br}=x_{c}+d_{r}$$

(19) $$y_{br}=y_{c}+d_{b}$$

The distribution rectangle concept is then used to estimate the influence of sharpness and color saliency factors in attracting viewers’ eyes. The sharpness and color saliency weights are computed as (20) and (21) where *R_I_* is the rectangle representing the distribution of pixel values in the image *I*, ¬*I* is the video inverted image of *I* and ‖R‖ represents the number of pixels in rectangle *R*.

(20) $$w_{sh}={\left(\frac{\|R_{\neg M^{sh}}\|}{\|R_{M^{sh}}\left\|+\right\|R_{M^{sh}}\cap R_{\neg M^{sh}}\|}\right)}^{2}$$

(21) $$w_{cs}={\left(\frac{\|R_{\neg M^{cs}}\|}{\|R_{M^{cs}}\left\|+\right\|R_{M^{cs}}\cap R_{\neg M^{cs}}\|}\right)}^{2}$$

The values of $w_{sh}$ and $w_{cs}$ reflect the influence of sharpness and color saliency in highlighting ROIs. The proposed ROI map is calculated as:

(22) $$M^{roi}=\frac{w_{sh}\times M^{sh}+w_{cs}\times M^{cs}}{w_{sh}+w_{cs}}$$

The binarized version $M_{b}^{roi}$ of the ROI map $M^{roi}$ is then obtained by applying the Otsu’s threshold to extract the ROIs. In Figure 7c,d, examples of the proposed ROI map and the binarized ROI map are shown.

#### 3.2.2. Deep Learning Based ROIE Method

**Figure 9:**
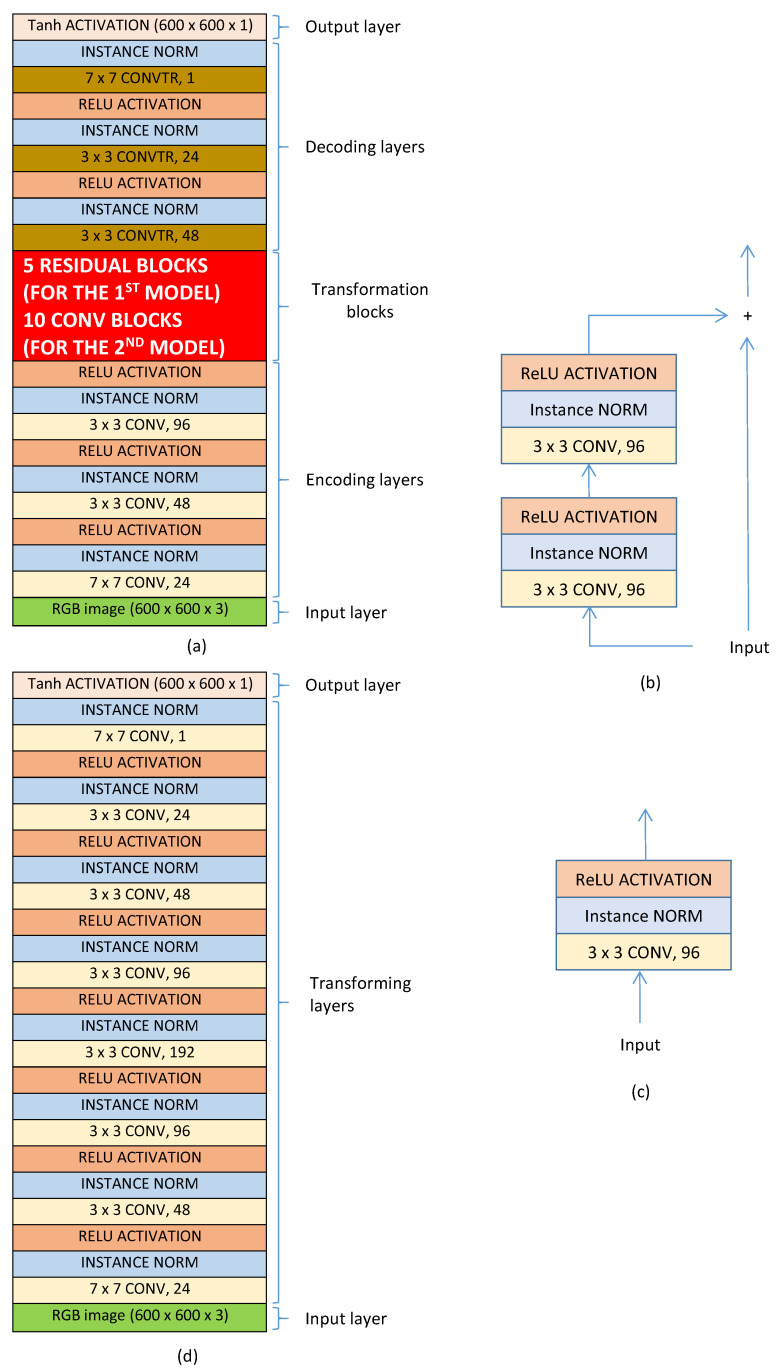
Structure of the three deep models: (**a**) structure of the two first models containing three main components: encoding component, transformation component and decoding component. (**b**) structure of a residual block. (**c**) structure of a convolutional block. (**d**) structure of the third model with convolutional blocks only.

Beside handcrafted approaches, deep learning based approaches might be a promising solution. In this part, three typical architectures are studied to find the best one for ROIE. The two first models are designed based on a well-known architecture with three main components: encoding, transformation and decoding components while the third one is designed based on a traditional architecture with convolutional blocks only. The structures of the three models are presented in Figure 9.

In the two first models, the first component contains three blocks of convolutional layers (see Figure 9a). In each block, a convolutional layer is connected to an instance normalization layer and it is activated by an ReLU function. The encoding component receives input color images of size 600×600 and passes the output to the transformation component. In the first model there are five residual blocks in the transformation component. The structure of a residual block is illustrated in Figure 9b with two blocks of convolutional layers. The transformed data is then concatenated with the input data to create the output of the block. In the second model, the transformation component contains 10 convolutional blocks (see the structure of a convolutional block in Figure 9c). The data transformed by the transformation component is passed through convolutional transpose layers of the decoding component and actived by a Tanh activation function to generate the binary ROI maps. The difference between the two first models is in the transformation components: the first model uses residual blocks while the second one uses convolutional blocks. On the contrary, the third model includes convolutional blocks only. There are eight convolutional blocks in the model and each block has a convolutional layer, an instance normalization layer and an ReLU activation layer (see Figure 9d). The numbers of kernels in the blocks are 24, 48, 96, 192, 96, 48, 24 and 1 respectively. The input layer and the output layer of the third model are similar to those of the two first models.

#### 3.2.3. Experiment and Results

##### Dataset and Setup

**Figure 10:**
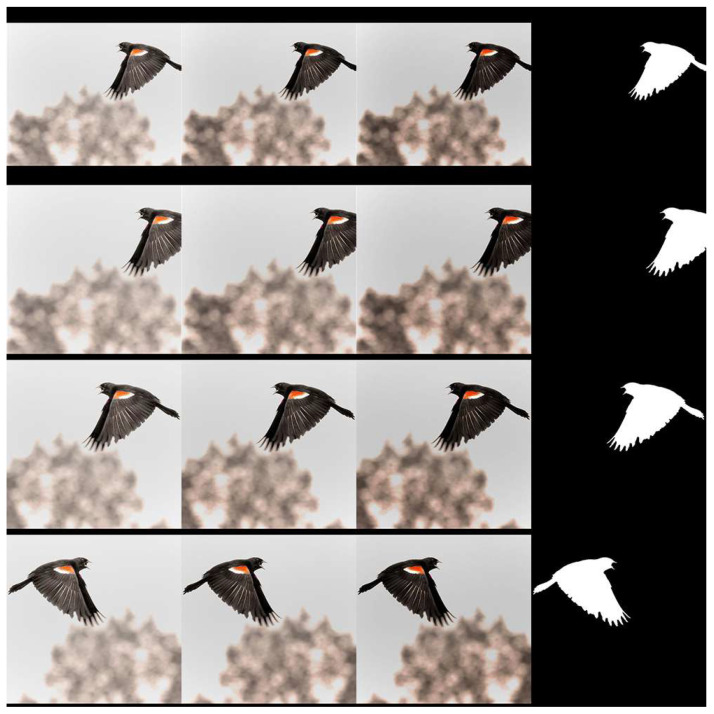
Examples of data augmentation. The three left columns contain the augmented versions while the last column shows the corresponding ROI ground truth.

1156 images (406 images from the CUHKPQ dataset [25] and 750 images from the Flickr.com website) are selected for the experiment. Following the ROI definition proposed in Section 2.3, each image is associated to a binary ground truth produced by the authors. The blur regions and unsalient color regions are considered as background (black regions in Figure 10) while sharp, high contrasted color regions are determined as ROIs (white regions in Figure 10). The proposed ROIE methods are evaluated on the dataset and they are compared with two methods based on sharpness information only (Aydin’s [5] and Tang’s [25] methods) and with two methods based on color contrast information only (Perazzi’s [27] and Zheng’s [28] methods).

In order to train and test the deep models, the dataset is divided into four parts (each part contains 289 images). The models are trained four times. Each time, only one part is used for the test while the others are considered for training. To increase the number images in training sets (because training deep models requires a big number of samples), a data augmentation process is applied. From an image, 200 augmented versions of size 600 × 600 are generated by flipping, re-scaling, padding, modifying brightness and shifting (see Figure 10). In the training phase, the chosen optimizer is the Adam optimizer and the loss function is the mean squared error function while the learning rate is set to $10^{-4}$.

For a given map in gray scale, pixel values range from 0 to 255, except for Tang’s ROI maps and ROI maps generated by the deep models (they are binary maps). The simplest way to compare those maps with the binary ground truth is to convert them into binary levels by applying a threshold. In this work, two thresholds have been considered. The first way is to use every threshold ranging from 0 to 255. The results are then used to form a precision recall curve. The Area Under Curve (AUC) is considered as the evaluation criterion. The second way is to choose a fixed threshold in which there are two options: Otsu’s threshold selected based on the gray histogram and the adaptive threshold defined as twice the mean of pixel values [50]. After performing the experiments, we conclude that applying Otsu’s threshold makes better results than applying the adaptive threshold so only results gained with Otsu’s threshold are presented in this section. The evaluation criteria with a fixed threshold are precision, recall, F-measure and IoU that are defined in Table 4. The range of a metric *X* within the 95% confidence interval [51,52] is described as $X\pm I_{X}$.

Five comparisons have been made to evaluate the methods. Firstly, the proposed sharpness estimation method is evaluated and compared with two methods based on sharpness information (Aydin’s and Tang’s methods). Secondly, the comparison between the proposed color saliency estimation method and two ROIE methods based on color contrast information (Perazzi’s and Zheng’s color saliency maps) is performed. The third one is to compare the proposed handcrafted ROIE method with the proposed sharpness estimation method and with the proposed color saliency method. The next comparison is for the three deep learning based methods to find the best model. The last comparison is between the handcrafted approach and the deep learning based approach.

##### Results and Discussion

**Figure 11:**
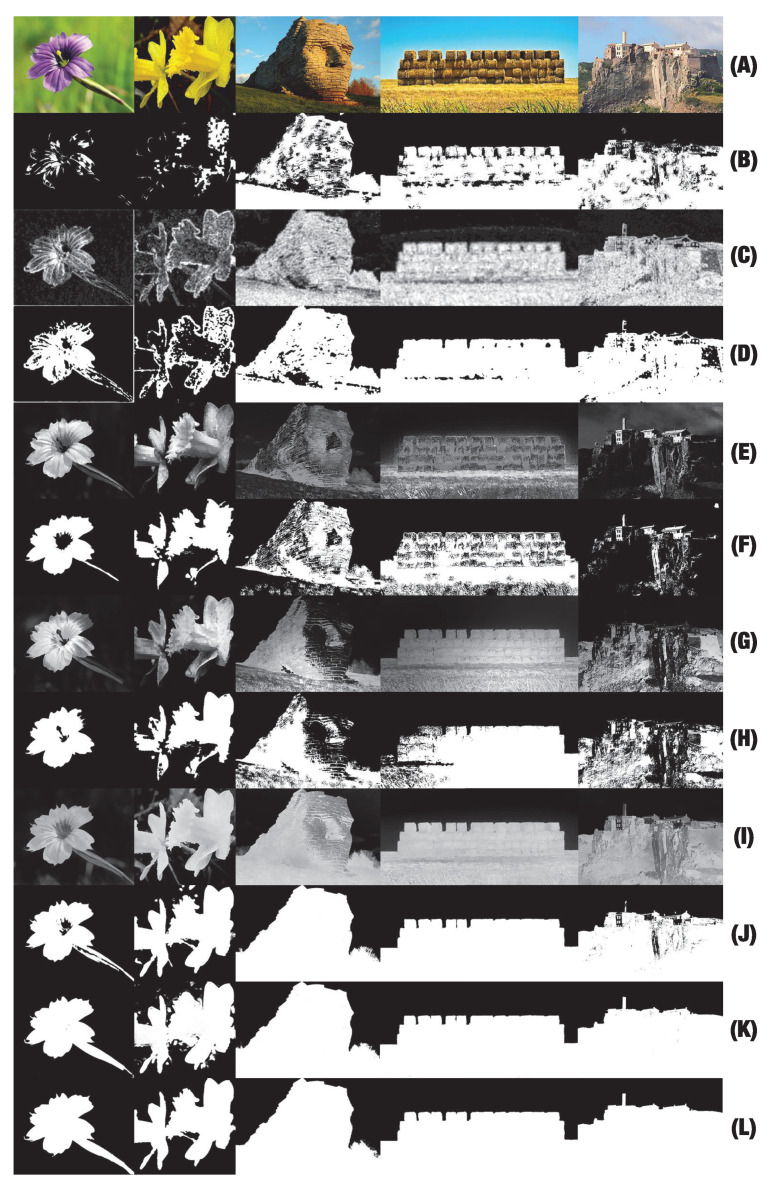
Examples of ROI maps. (**a**) Original images. (**b**) Tang’s [25] sharpness maps. (**c**,**d**) Aydin’s [5] clearness maps and the binarized versions of them. (**e**,**f**) Perazzi’s [27] color saliency maps and the binarized versions of them. (**g**,**h**) Zheng’s [28] color saliency maps and the binarized versions of them. (**i**,**j**) Handcrafted ROI maps based on both sharpness and color information and the binarized versions of them. (**k**) ROI maps generated by the first deep model. (**l**) ground truth.

**Figure 12:**
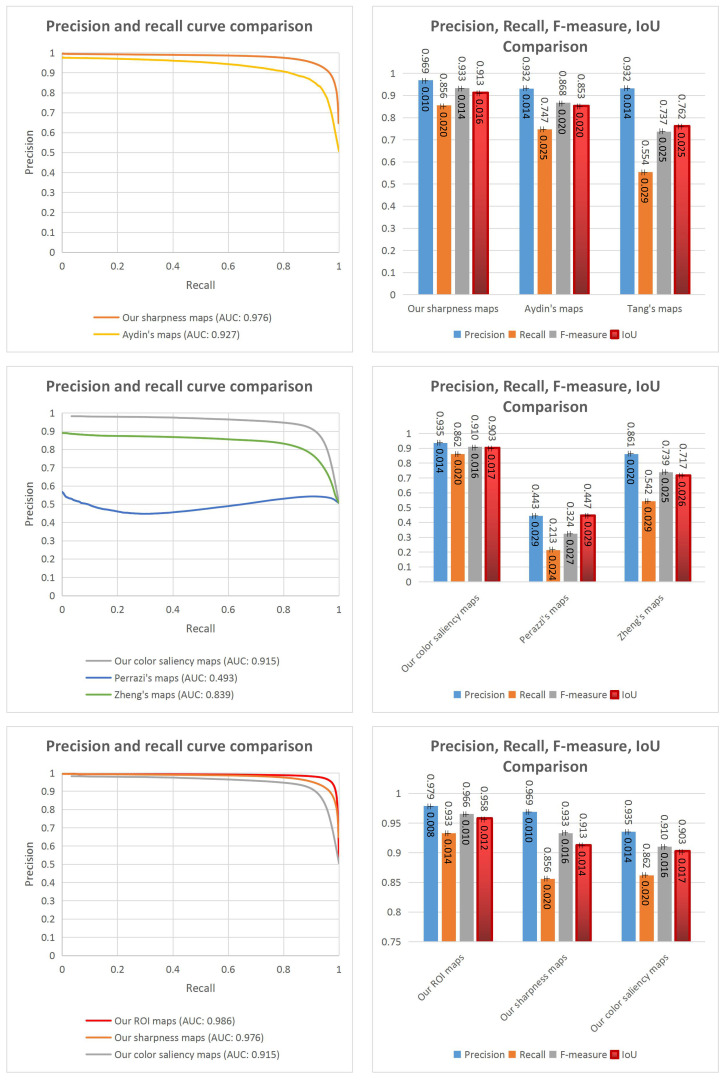
Evaluations for ROI maps. First row: Evaluations for the proposed sharpness estimation method, Aydin’s method and Tang’s method (Tang’s ROI maps are binary maps so it is not necessary to consider their precision and recall curve). Second row: Evaluations for the proposed color saliency estimation method, Perazzi’s method and Zheng’s method. Third row: Evaluations for our handcrafted ROIE method, sharpness estimation method and color saliency estimation method.

**Figure 13:**
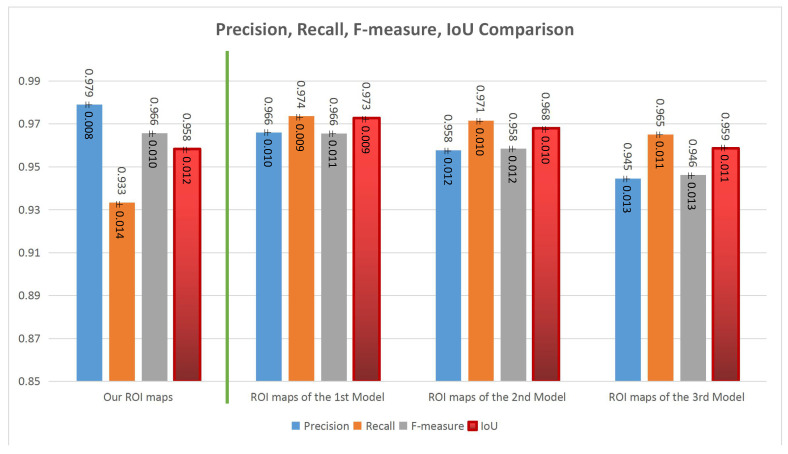
Evaluations for our handcrafted ROIE method (on the left side) and our deep models (on the right side).

Examples of different ROI maps are shown in Figure 11. Comparing the results in binary scale (see Figure 11b,d,f,h,j,k), the results at rows (j) and (k) representing our ROIE methods are better since they are smoother, have more precise details and less background noise than other results. The results of Tang’s method do not seem precise in the case of the two first columns since their results mainly focus on few sharp details of the two close-up images. The results for large field images seem better than those of close-up images. The results of Aydin’s method look better than those of Tang’s method but they are still not good enough. The results of Perazzi’s and Zheng’s methods at the two first columns of row (e) and row (g) are better than those of Aydin’s and Tang’s methods but the results are not really good for large field images where sharpness factor is dominant. The main superiority of our methods is the high accuracy in both cases when photographers consider either sharpness or color saliency to define ROIs. The evaluations for the methods are presented in Figure 12 and Figure 13.

Firstly, the comparison between our sharpness estimation method and the two ROIE methods based on sharpness information is shown in the first row of Figure 12. Looking at the precision recall curves, the AUC value of the proposed method is better than that of Aydin’s method (0.976 against 0.927). The column chart shows that the highest values of precision, recall, F-measure and IoU belong to our method around 0.969±0.010, 0.856±0.005, 0.933±0.014, 0.913±0.016 respectively.

Secondly, the second row of Figure 12 shows the comparison between our color saliency estimation method and the two ROIE methods based on color contrast information. The charts indicate that the highest values of AUC (0.915), precision (0.935±0.014), recall (0.862±0.020), F-measure (0.910±0.016) and IoU (0.903±0.017) are associated to our method. The cause of the bad results of Perazzi’s method might be the differences between their color saliency definition and our color saliency definition since Perazzi et al. mostly focus on color contrast between all regions so regions having the most different colors are considered as the regions with the highest color saliency levels. In Zheng’s method, they consider initially the colors of the center regions as salient so the results of Zheng’s method are better than those of Perazzi’s method.

The third comparison is for our sharpness maps, our color saliency maps and our handcrafted ROI maps. Looking at the graphs in the third row of Figure 12, the results of the proposed ROIE method are better than those of the sharpness estimation method and the color saliency estimation method with the highest AUC (0.986), precision (0.979±0.008), recall (0.933±0.014) and F-measure (0.966±0.010) and IoU (0.958±0.012) values. It proves the efficiency of combining sharpness and color information to extract ROIs.

The comparison between the three proposed deep models is presented on the right side of Figure 13. Generally, all the three models have good performances. The first model (with encoding, transformation and decoding components using residual blocks) has the highest performance around 0.966±0.010, 0.974±0.009, 0.966±0.011 and 0.973±0.009 for precision, recall, F-measure and IoU values respectively. It reflects that the architecture with the three main components is the best one and residual blocks seem better than convolutional blocks in this case.

Comparing the handcrafted ROIE method and the deep learning based method, the precision and F-measure values of the two methods are almost the same but the deep model has higher recall values (0.974±0.010) and a better balance between precision, recall and F-measure than those of the handcrafted ROIE method. Generally, the two proposed methods have impressive results and the results of the deep learning based method are slightly better than those of the handcrafted method (IoU values: 0.973±0.009 versus 0.958±0.012).

In this part, we point out that sharpness only or color saliency only are not enough to precisely define ROIs (regions attracting viewers’ eyes) while the combination of the two factors improves the performances. This ROIE task has been studied with both handcrafted and deep learning based approaches. They have been tested and compared with four other ROIE methods on a dataset containing 1156 images with the ROI ground truth. The gained results are quite good for both proposed methods but the results of deep learning based method are slightly better so the deep learning based ROIE method is going to be considered in the next sections. ROIE is a preparation step before computing ROI features and background features from the corresponding regions. The influence of ROI features and background features in IAA is going to be estimated in the next sections.

### 3.3. Conclusions

ROIE and LCIC are preliminary steps before performing IAA. Firstly, starting with the results of LCIC, IAA based on the classification is studied and it is then compared with IAA without image classification to evaluate the influence of prior image classification in IAA. Secondly, the roles of global features (extracted from the whole image without ROIE) and local features (ROI and background features computed from ROIE) in IAA for large field images only and IAA for close-up images only are studied to clarify the role of prior ROIE in IAA.

## 4. Feature Definition

Features in this section are defined for the purpose of evaluating the influence of prior ROIE and LCIC in IAA so three feature sets computed on the whole image, ROIs and background are built for General IAA (GIAA: IAA for all kind of images), Large field IAA (LIAA: IAA for large field images only) and Close-up IAA (CIAA: IAA for close-up images only). Additionally, rules of photographic art are the main inspirations for designing aesthetic features either on the whole images or on local regions. However, aesthetic is an abstract concept depending on individual feelings and subjective opinions so it is not easy to describe, explain or modelize all aesthetic aspects and aesthetic characteristics. Learned features could be a good solution for this problem. Therefore, both handcrafted and deep learning based feature approaches are considered in this study.

### 4.1. Handcrafted Feature Definition

Starting with a large handcrafted feature set built from common handcrafted features (computed from the whole image, ROIs and background based on hue, saturation, brightness, red, green and blue channels, sharpness, color saliency and contrast information) appearing in different researches [5,24,37,38,39], the feature selection process presented in Section 3.1.2 is applied with 18,048 images coming from various image categories, 800 large field images and 800 close-up images to build three aesthetic feature sets for GIAA, LIAA and CIAA respectively. Feature vector $F_{h}^{a}$ contains the 24 most relevant features for GIAA while two feature vectors: $F_{h}^{l}$ containing the 21 most relevant features and $F_{h}^{c}$ containing the 23 most relevant features are considered for LIAA and CIAA respectively. The details of the three feature sets are presented in Table 5, Table 6 and Table 7.

### 4.2. Learned Feature Definition

Even though the most relevant features are selected from many handcrafted features, it is possible that some aesthetic aspects have not been considered so the idea here is to use deep learning based approach to tackle the problem.

#### 4.2.1. Learned Features for GIAA

**Figure 14:**
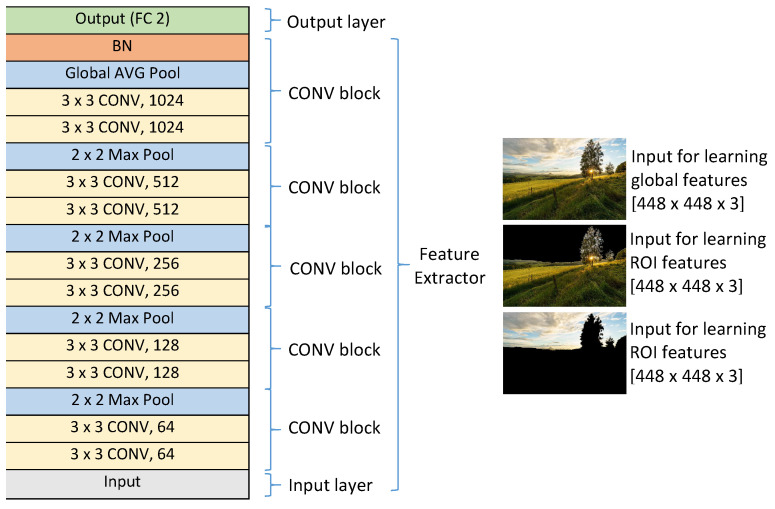
General structure of the models learning aesthetic features from the whole image, ROIs and background.

**Figure 15:**
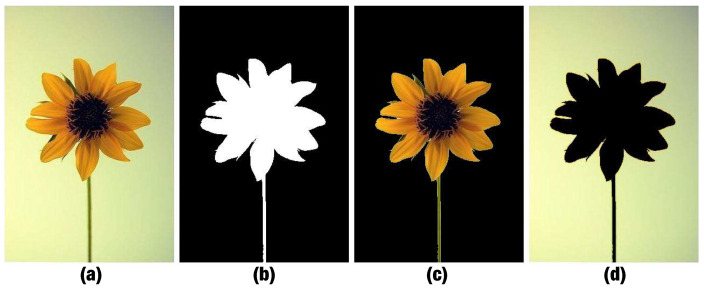
Examples of the two generated versions based on ROIE. (**a**) The original image. (**b**) The ROI map. (**c**) The first version. (**d**) The second version.

Three deep CNNs are used to learn aesthetic features from the whole image, ROIs and background. A typical CNN architecture with an input layer, an output layer and five convolutional blocks (see the general architecture of the three CNNs in Figure 14) is chosen. Each convolutional block has two convolutional layers and a pooling layer. The numbers of kernels in those blocks are 64×2, 128×2, 256×2, 512×2, 1024×2 respectively (there are two convolutional layers in each block). In the four first blocks, max pooling layers are used while a global average pooling layer is used in the last block and it is connected to a batch normalization layer before passing data to the output layer. The output layer contains two output neurons corresponding to the two classes: high aesthetic image and low aesthetic image while the input layer receives color images of size 448 × 448 (448×448×3). From an input image, two transformed versions are generated (see Figure 15). In the first one, values of all pixels belonging to the background are set to 0 while all values of pixels in the ROIs are kept the same as the corresponding pixels in the input image (see Figure 15c, this is for ROI feature learning). In contrast, all pixel values of the ROIs in the second version are set to 0 while all background pixel values are kept the same as the corresponding pixels of the input image (see Figure 15d, this is for the background feature learning). The first CNN considers the original image as the input of the model to learn aesthetic features from the whole image while the second and the third models consider the first and the second transformed versions as the input to learn aesthetic features from ROIs and background respectively.

Those deep CNNs are trained on 9024 high aesthetic images and 17,666 low aesthetic images coming from the CUHKPQ dataset [25]. Those models require a very big number of samples so a data augmentation method is applied. Similarly to the data augmentation in Section 3.2.3, from the original version of any low aesthetic image, 100 transformed versions of size 448×448 (this size is not too small to affect image aesthetic so the aesthetic labels of the transformed versions are kept the same as those of the original versions) are generated by re-scaling, padding, cropping and shifting while 200 transformed versions of size 448×448 are generated from the original version of any high aesthetic image by re-scaling, padding, cropping, shifting and flipping (flipped versions are added to balance the number of images in the two classes). Thus, the numbers of high and low aesthetic image in the training set are 1,804,800 (9024×2×100) and 1,766,600 (17,666×100) respectively (the labels of transformed versions are set the same as the label of the original version). If the last layer of each model is removed, the three models become three feature extractors computing 1024 aesthetic features learned from the whole image $F_{l}^{g}$, 1024 aesthetic features learned from ROIs $F_{l}^{r}$ and 1024 aesthetic features learned from background $F_{l}^{b}$ respectively.

In order to compare with the handcrafted feature set $F_{h}^{a}$, the 24 (the same number as the number of handcrafted features for GIAA) most relevant features ($F_{l}^{a}$) are selected for GIAA based on feature relevance computed by the Relief method.

#### 4.2.2. Learned Features for LIAA and CIAA

**Figure 16:**
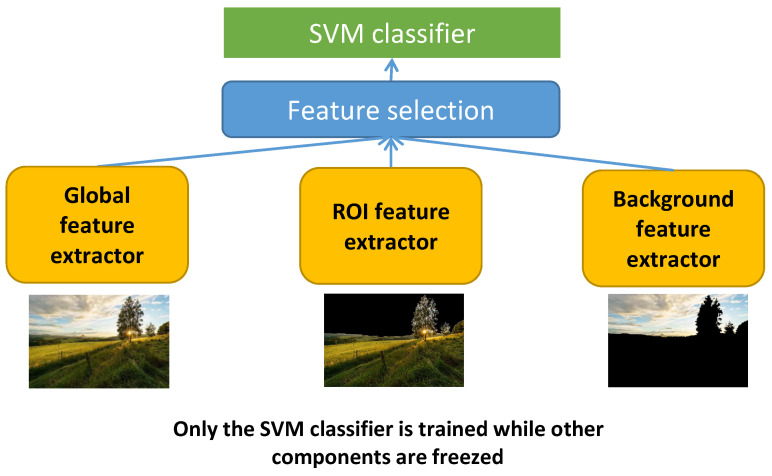
Models using transferred features for LIAA, CIAA and GIAA.

In general, learning features directly from images often requires many samples. Although there are some datasets with aesthetic labels for all kinds of images, an aesthetic dataset for only large field images and close-up images is not available so we do not have enough data to learn aesthetic features directly. Transfer learning could be a good choice in this case. Starting with the aesthetic features $F_{l}^{a^{*}}=F_{l}^{g}\cup F_{l}^{r}\cup F_{l}^{b}$ learned in the previous part, there are 3072 aesthetic features including 1024 global features ($F_{l}^{g}$: features learned from the whole image), 1024 ROI features ($F_{l}^{r}$: features learned from the ROIs) and 1024 background features ($F_{l}^{b}$: features learned from the background). Those features are learned to perform GIAA for all kinds of images and we want to transfer them to focus on large field images only and close-up images only. The main idea in this case is presented in Figure 16, the deep models without the last layer are considered as feature extractors to compute global features, ROI features and background features. Those computed features of large field images and close-up images only are considered as input to train new IAA models for large field images and close-up images respectively. There is a feature selection step in the process because there are 3072 learned features while the number of large field and close-up images used in this work is 2400 (1200 large field images and 1200 close-up images). It seems that the higher number of features could lead to an overfitting so it is necessary to reduce the number of learned features. The 21 most relevant features ($F_{l}^{l}$) are selected from the 3072 learned aesthetic features to perform the LIAA task (the same number as the number of handcrafted features for LIAA) and the 23 most relevant features ($F_{l}^{c}$) are selected for the CIAA task (the same number as the number of handcrafted features for CIAA) based on feature relevance computed by using the Relief method.

## 5. Image Aesthetic Assessment: Prior Image Classification or not Prior Image Classification?

The main question of this section are “Is it worthy to proceed to LCIC before IAA?”. In order to answer the question, IAA based on the results of the prior LCIC is compared with IAA without prior LCIC. In this section, we use two approaches: handcrafted features and learned features to answer also the question “How efficient handcrafted features and learned features are in IAA?”.

### 5.1. Dataset and Setup

**Figure 17:**
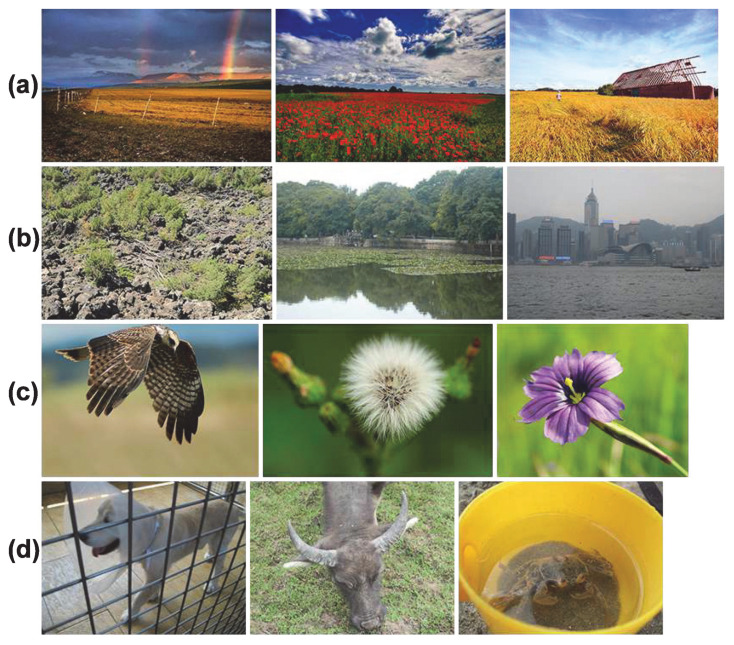
Examples of high and low aesthetic images: (**a**) high aesthetic large field images, (**b**) low aesthetic large field images, (**c**) high aesthetic close-up images, (**d**) low aesthetic close-up images.

A part of the CUHKPQ dataset is extracted to form an aesthetic dataset with large field and close-up images only. The CUHKPQ dataset is collected mainly from DPChallenge.com website and from some other sources. All the images are labelled as high or low aesthetic. A photo is indicated as high/low aesthetic if there are at least eight of the ten viewers having the same opinion about the image aesthetic [25]. Viewers’ aesthetic perception does not depend on distortions, artifacts or degradation, … but it is affected by perspective of visual aesthetic, photography technique: simplicity, realism, composition, lighting, color arrangement, camera settings, topic emphasis, … There are seven categories of the CUHKPQ dataset including animal, plant, static, architecture, landscape, human and night. Large field images are selected from the architecture and landscape categories while close-up images are extracted from the animal, plant, static and human categories (see examples in Figure 17). The extracted part contains 1200 large field images and 1200 close-up images in which 50% of the images in each category are labelled as high aesthetic and the others are labelled as low aesthetic by humans. In order to train an IAA model assessing image aesthetic automatically, 800 large field images and 800 close-up images are selected for training and the remains (400 large field images and 400 close-up images) are used for testing.

There are two main experiments in this section. The first one is to perform IAA without prior image classification using the feature vectors $F_{h}^{a}$ and $F_{l}^{a}$ (for all kinds of images). The second experiment is to perform the IAA with prior LCIC using the feature vectors $F_{h}^{l}$, $F_{l}^{l}$ (features for large field images only) for LIAA and using feature vectors $F_{h}^{c}$, $F_{l}^{c}$ (features for close-up images only) for CIAA. Those experiments are performed to answer two questions: “Is it worthy to perform prior image classification for IAA?” and “How efficient handcrafted features and learned features are in IAA?”. Additionally, IAA with and without LCIC are performed using two handcrafted image aesthetic feature sets: Suran’s [10] and Aydin’s [5] sets ($F_{h}^{a_{1}}$ and $F_{h}^{a_{2}}$ respectively).

An SVM classifier is trained based on those feature vectors to indicate an image as high or low aesthetic. The parameters for the SVM are set as C=0.5, γ=auto. Different kernels including Poly, Linear, RBF and Sigmoid are tested and only the best results (with an RBF kernel) are presented.

The evaluation criteria of the experiments are presented in Table 8. Accuracy (*A*), a popular evaluation criterion for classification tasks is the main criterion for the evaluation while confidence interval ($I_{a}$), the lower bound of the accuracy ($A_{l}$) and the upper bound of the accuracy ($A_{u}$) reflect the range of the accuracy. The experiments have been conducted on a PC equipped with an Intel(R) Xeon(R) W-2104 CPU 3.20 GHz, 31.7 GB memory and GPU NVIDIA Quadro P400.

### 5.2. Results and Discussion

The results of IAA with and without image classification are presented in Table 9. Either with handcrafted features or learned features, the performances of IAA with prior image classification are better than the results of IAA without prior image classification (0.940±0.023, 0.925±0.026 for LIAA, CIAA versus 0.921±0.018 for GIAA with learned features; 0.913±0.028, 0.843±0.036 for LIAA, CIAA versus 0.785±0.028 for GIAA with our handcrafted features; 0.880±0.031, 0.860±0.034 for LIAA, CIAA versus 0.845±0.025 for GIAA with Suran’s features; 0.878±0.032, 0.833±0.037 for LIAA, CIAA versus 0.800±0.028 for GIAA with Aydin’s features). It appears that performing LIAA and CIAA separately using different aesthetic features could enhance the IAA performance (not only our IAA methods but also other IAA methods). As guessed, since large field images and close-up images are two image categories having opposite photographic rules such as the composition, depth of field, focus, … so the criteria for LIAA and CIAA are not the same. Considering the relations between the two feature sets $F_{l}^{l}$ (features for LIAA) and $F_{l}^{c}$ (features for CIAA), they are really different since there are only three overlapping features between the two feature sets. Thus, the aesthetic quality of the two image categories should be assessed separately using different criteria. As a result, it is worthy to proceed to LCIC before IAA whatever the used method for IAA.

Moving to the second question “How efficient handcrafted features and learned features are in IAA?”, in both cases (GIAA and LIAA/CIAA), learned features are better than handcrafted features. More specifically, in the case of GIAA, the performance with learned features is 0.921±0.018 while the results with handcrafted features are 0.785±0.028, 0.845±0.025 and 0.800±0.028 for our features, Suran’s features and Aydin’s features respectively. Similarly, in the case of IAA for a particular image category (large field images only or close-up images only), the results of LIAA and CIAA with learned features and our handcrafted features, Suran’s features, Aydin’s features are 0.940±0.023 versus 0.913±0.028, 0.880±0.031, 0.878±0.032 and 0.925±0.026 versus 0.843±0.036, 0.860±0.034, 0.833±0.037 respectively. As mentioned in the previous part, image aesthetic is an abstract concept depending on human perception and individual feeling so understanding and defining all aesthetic aspects are not easy. However, handcrafted aesthetic features are designed based on aware aesthetic aspects so it is impossible to design handcrafted features representing unconscious aesthetic aspects. On the contrary, deep models can learn complex and non visible aesthetic features so we can find some similarities between image aesthetic notion and learned features. It could be the reason why the results with learned features are better than the ones with handcrafted features. According to those results, the final conclusion is achieved: learned features are very efficient and they are better than handcrafted features for IAA. The following section focuses on learned features only because of their higher performances.

## 6. Image Aesthetic Assessment: Prior Region Segmentation or not Prior Region Segmentation?

The main goal of this section is to evaluate the role of ROIE in IAA. The role of ROIs is not always the same for each image so the influence of ROIE in IAA for a particular image category (large field images only or close-up images only) is going to be considered. The two learned feature sets $F_{l}^{l}$ (for LIAA) and $F_{l}^{c}$ (for CIAA) presented in the previous section are analyzed to estimate the influence of ROIE in IAA.

### 6.1. Dataset and Setup

There are two main tasks in this part. Firstly, the distribution of ROI and background features (RB features) in each feature set ($F_{l}^{l}$ and $F_{l}^{c}$) is analyzed to have an overall view about the role of ROIE in LIAA and CIAA. Secondly, IAA using RB features is compared with IAA using global features and with IAA using both global and RB features to estimate how ROIE affects IAA.

The experiments of LIAA and CIAA using the feature sets $F_{l}^{l}$ and $F_{l}^{c}$ respectively are performed on 1200 large field images and 1200 close-up images (the same as the dataset of the experiments of LIAA and CIAA in the previous section) in which 800 large field images and 800 close-up images (50% of the images in each category are labelled as high aesthetic and the others are labelled as low aesthetic) are used for training while the remains are used for testing.

As done before, the parameters of the classifiers are set as C=0.5, γ=auto and different kernels are tested and only the best results are presented. The main evaluation criterion is the accuracy. The range of the accuracy is presented by the confidence interval, the lower bound of the accuracy and the upper bound of the accuracy.

### 6.2. Results and Discussion

Firstly, Table 10 shows the number of global features and RB features (ROI features and background features) in each feature set ($F_{l}^{l}$ and $F_{l}^{c}$). It appears that the role of ROIE in IAA is not the same for all image categories. In the case of close-up images, ROIE has the most significant role in IAA since the number of RB features in $F_{l}^{c}$ is the highest (five features). In contrast, there is no RB feature in the feature set $F_{l}^{l}$ for LIAA. The reason probably is that the content of a large field photo is a large scene (as the name of the category) so viewers often pay attention to the whole large scene including both ROIs and background. Therefore, the influence of ROIE in LIAA is not significant so LIAA is skipped in the next analysis.

Secondly, the evaluations of global features ($F_{g}^{c}$: global features in $F_{l}^{c}$) and RB features ($F_{rb}^{c}$: ROI and background features in $F_{l}^{c}$) for CIAA are presented in Table 11. The results are quite interesting since with only five RB features, the obtained classification accuracy is very impressive (0.868±0.033). The combination of five RB features and 18 global features helps increasing the IAA performance from 0.908±0.028 to 0.925±0.026.

Two additional image aesthetic feature sets are considered to validate the role of ROIE in IAA. The first one is Suran’s feature set ($F_{h}^{a_{1}}$) [10] containing 10 global features ($F_{g}^{a_{1}}$) and 28 RB features ($F_{rb}^{a_{1}}$) in which Suran’s ROIs are defined as the three largest segments of a given image. The second one is Aydin’s feature set ($F_{h}^{a_{2}}$) [5] including four global features ($F_{g}^{a_{2}}$) and one RB feature ($F_{rb}^{a_{2}}$) in which Aydin’s ROIs are defined as the sharp and clear regions of a given photo. The results of the LIAA and CIAA based on those feature sets are presented in Table 12. Considering results with Aydin’s features where ROIs are defined as sharp and clear regions, it is obvious that the LIAA performance with the RB feature is very bad at 0.540±0.049 while the LIAA performance with global features is even better than that with both global and RB features (0.888±0.031 versus 0.878±0.032). It means that RB features has an insignificant role in LIAA. In contrast, the performance of CIAA with only one RB feature is much better than that with four global features (0.818±0.038 versus 0.740±0.043) and the combination of $F_{g}^{a_{2}}$ and $F_{rb}^{a_{2}}$ helps improving the CIAA performance to 0.833±0.037. Those results demonstrate a significant role of RB features and ROIE in CIAA. Considering results with Suran’s features, it appears that RB features do not help improving LIAA and CIAA performances since the results with the global features only are approximately the results with both global and RB features (0.875±0.032 versus 0.888±0.031 for LIAA and 0.853±0.035 versus 0.860±0.034 for CIAA). It could be explained that Suran’s ROI definition is too simple (top three largest segments) so extracted ROIs are not precise enough to improve the performance of IAA. Thus, it is obvious that extracting precise ROIs has an important role in improving IAA performance.

The background of close-up images is often blur to highlight the main close-up object regions (sharp regions with high contrasted colors - ROIs) so viewers often pay more attention on ROIs. It explains why ROIs have significant influence on aesthetic quality of close-up images. According to those results, it appears that it is worthy to extract ROIs before assessing aesthetic quality of close-up images.

In general, the role of ROIE in IAA is various since the influence of ROIE in IAA for large field images is insignificant while ROIE helps improving the IAA for close-up images. The answer to the question “IAA: prior region segmentation or not?” might depend on the considered situation.

## 7. Conclusions

**Figure 18:**
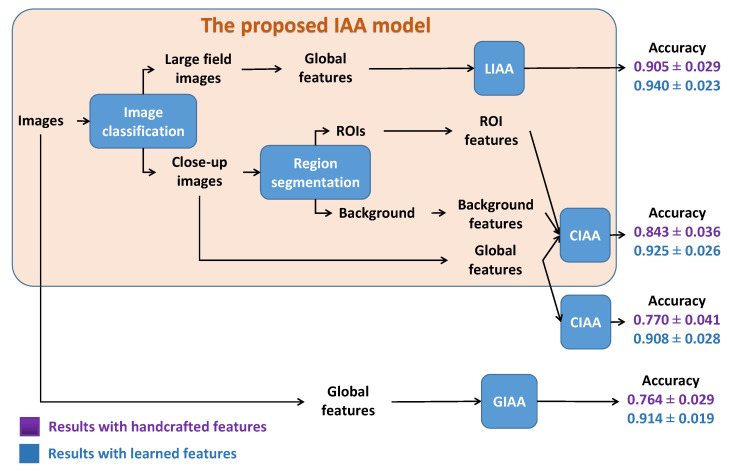
Proposed algorithm for IAA.

In this paper, the main works were to study IAA with image classification or region segmentation. Firstly, the experimental results prove that classifying images before performing the IAA can enhance the IAA performance. Secondly, performing prior ROIE before IAA or not depends on image type. Based on the obtained results, we propose an IAA model based on LCIC and ROIE. Figure 18 presents the idea of the proposed model. Images are first classified as large field images and close-up images. Then, large field images are assessed as high or low aesthetic quality by a classifier based on global features only. On the contrary, ROIs and background are extracted from close-up images to compute ROI features and background features. Those features are then combined with global features to make the distinction between high and low aesthetic close-up images. Figure 18 also shows the performances of the model compared with IAA without image classification and region segmentation. Firstly, it appears that image classification helps improving the IAA performances by assessing aesthetic quality of large field images and close-up images separately. Secondly region segmentation helps for CIAA especially in the case of handcrafted features. Both handcrafted features and learned features have been considered in this study and unsurprisingly learned features are more efficient. Besides, two pre-processing phases for IAA: ROIE and LCIC have been studied. For ROIE, the combination of sharpness and color factors makes a more precise definition of ROIs. Both the handcrafted and deep learning based methods are good but the results with the deep learning based method is slightly better. For LCIC, EXIF features are interesting because of their simplicity and their efficiency but learned features are the best choice for LCIC with the highest performance and the acceptable computational time.

## Figures and Tables

**Table 1:** Overview of the proposed handcrafted features for LCIC. $R_{1},R_{2},\ldots R_{9}$ are the regions split by the rule of thirds (see the top right photo in Figure 5).

Features	Formula
Sharpness features	$f_{1}:$ mean of gradient values in $R_{2}$
	$f_{2}:$ mean of gradient values in $R_{7}$
	$f_{3}:$ mean of gradient values in $R_{9}$
	$f_{4}:$ standard deviation of gradient values in $R_{5}$
	$f_{5}:$ gradient contrast between $R_{1}$ and $R_{7}$
	$f_{6}:$ gradient contrast between $R_{2}$ and $R_{8}$
	$f_{7}:$ gradient contrast between $R_{3}$ and $R_{9}$
	$f_{8}:$ standard deviation of gradient values in the whole image
Color features	$f_{9}:$ brightness contrast between $R_{1}$ and $R_{7}$
	$f_{10}:$ brightness contrast between $R_{2}$ and $R_{8}$
	$f_{11}:$ brightness contrast between $R_{3}$ and $R_{9}$
	$f_{12}:$ color contrast between $R_{1}$ and $R_{7}$
	$f_{13}:$ color contrast between $R_{2}$ and $R_{8}$
	$f_{14}:$ color contrast between $R_{3}$ and $R_{9}$
ROI/background features	$f_{15}:$ proportion of ROI pixels in $R_{2}$
	$f_{16}:$ proportion of ROI pixels in $R_{7}$
	$f_{17}:$ proportion of ROI pixels in $R_{9}$
	$f_{18}:$ mean of gradient values in ROIs
	$f_{19},f_{20},f_{21}:$ relations between ROIs and background

**Table 2:** Overview of evaluation criteria for LCIC. z=1.96 for 95% confidence interval and the number of samples *N* is 800

Evaluation Criteria	Formula
Accuracy	$A=\frac{TP+TN}{TP+FP+TN+FN}$
Confidence interval of accuracy	$I_{a}=z\times \sqrt{\frac{(1-A)\times A}{N}}$
Feature computational time	$T_{F}$
Classification time	$T_{C}$
Total computational time	$T_{T}=T_{F}+T_{C}$

**Table 3:** LCICs using EXIF features, handcrafted features and learned features.

**LCIC Using the Four EXIF Features**				
$A\pm I_{a}$ = 0.878±0.023	Without the GPU	$T_{F}$ = 1 ms	$T_{C}$ = 1 ms	$T_{T}$ = 2 ms
**LCIC Using the 21 Handcrafted Features**				
$A\pm I_{a}$ = 0.873±0.023	Without the GPU	$T_{F}$ = 30 ms	$T_{C}$ = 1 ms	$T_{T}$ = 31 ms
LCIC using	Wang’s feature set (105 features)		Zhuang’s feature set (257 features)	
	$A\pm I_{a}$ = 0.774±0.029		$A\pm I_{a}$ = 0.854±0.024	
**LCIC Using the 925 Most Relevant VGG16 Features**				
$A\pm I_{a}$ = 0.989±0.007	Without the GPU	$T_{F}$ = 434 ms	$T_{C}$ = 2 ms	$T_{T}$ = 436 ms
	With the GPU	$T_{F}$ = 16 ms	$T_{C}$ = 2 ms	$T_{T}$ = 18 ms
**LCIC Using the 21 Most Relevant VGG16 Features**				
$A\pm I_{a}$ = 0.981±0.009	Without the GPU	$T_{F}$ = 434 ms	$T_{C}$ = 1 ms	$T_{T}$ = 435 ms
	With the GPU	$T_{F}$ = 16 ms	$T_{C}$ = 1 ms	$T_{T}$ = 17 ms
**LCIC Using the Four Most Relevant VGG16 Features**				
$A\pm I_{a}$ = 0.975±0.011	Without the GPU	$T_{F}$ = 434 ms	$T_{C}$ = 1 ms	$T_{T}$ = 435 ms
	With the GPU	$T_{F}$ = 16 ms	$T_{C}$ = 1 ms	$T_{T}$ = 17 ms

**Table 4:** Evaluation criteria of ROI detection methods: Precision, Recall, F-measure and Intersection over Union where TP,FN,FP,TN are a number of pixels. $ROI_{P}$, $ROI_{G}$ are predicted ROIs and ROIs according to the ground truth. $BG_{P}$, $BG_{G}$ are predicted background and background according to the ground truth. β=0.3, N=1156.

Evaluation Criteria of ROI Detection Methods			
Precision	$Pr=\frac{TP}{TP+FP}$	$I_{Pr}=z\times \sqrt{\frac{Pr\times (1-Pr)}{N}}$	
Recall	$Re=\frac{TP}{TP+FN}$	$I_{Re}=z\times \sqrt{\frac{Re\times (1-Re)}{N}}$	
F-measure	$F_{\beta }=\frac{(1+\beta^{2})\times Pr\times Re}{\beta^{2}\times Pr+Re}$	$I_{F_{\beta }}=z\times \sqrt{\frac{F_{\beta }\times \left(1-F_{\beta }\right)}{N}}$	
Intersection over Union	$IoU=\frac{TP}{TP+FP+FN}$	$I_{IoU}=z\times \sqrt{\frac{IoU\times (1-IoU)}{N}}$	
$TP=ROI_{P}\cap ROI_{G}$	$FP=ROI_{P}\cap BG_{G}$	$FN=BG_{P}\cap ROI_{G}$	$TN=BG_{P}\cap BG_{G}$

**Table 5:** Overview of the proposed handcrafted features $F_{h}^{a}$ for GIAA.

Features	Formula
Global features	$f_{1}$: mean of gradient values
	$f_{2}$: mean of brightness values
	$f_{3}$: standard deviation of brightness values
	$f_{4}$: number of main brightness bins (brightness
	range is split into 64 bins)
	$f_{5}$: mean of saturation values
	$f_{6}$: standard deviation of saturation values
	$f_{7}$: kurtosis of saturation values
	$f_{8}$: standard deviation of hue values
	$f_{9}$: number of main hue bins (hue range is
	split into 64 bins)
	$f_{10}$: number of main colors
	$f_{11}=\sqrt{\sigma_{Re}^{2}+\sigma_{Gr}^{2}+\sigma_{Bl}^{2}}$
	$\sigma_{Re}$, $\sigma_{Gr}$ and $\sigma_{Bl}$ are standard deviation of red, green
	and blue values
	$f_{12}$, $f_{13}$: coordinate of the center point determined
	by gradient values
	$f_{14}$, $f_{15}$: coordinate of the center point determined
	by saturation values
	$f_{16}$, $f_{17}$: coordinate of the center point determined
	by brightness values
ROI and background features	$f_{18}$: number of main hue bins of ROIs
	$f_{19}$: mean of gradient values of ROIs
	$f_{20}$: brightness contrast between ROIs and background
	$f_{21}$: mean of gradient values of background
	$f_{22}$: mean of brightness values of background
	$f_{23}$: number of main saturation bins of background
	$f_{24}$: number of main hue bins of background

**Table 6:** Overview of the proposed handcrafted features $F_{h}^{l}$ for LIAA.

Features	Formula
Global features	$f_{1}$: mean of gradient values
	$f_{2}$: standard deviation of gradient values
	$f_{3}$: mean of brightness values
	$f_{4}$: standard deviation of brightness values
	$f_{5}$: mean of saturation values
	$f_{6}$: standard deviation of saturation values
	$f_{7}$: colorfulness
	$f_{8}$: min distance to intersection points (based on the
	rule of thirds) determined by sharpness values
	$f_{9}$: min distance to intersection points (based on the
	rule of thirds) determined by color saliency values
	$f_{10}$: min distance to intersection points (based on the
	rule of thirds) determined by brightness values
	$f_{11}=\min\left(f_{8},f_{9},f_{10}\right)$
ROI and background features	$f_{12}$: mean of gradient values of ROIs
	$f_{13}$: mean of color saliency values of ROIs
	$f_{14}$: mean of saturation values of ROIs
	$f_{15}$: mean of brightness values of ROIs
	$f_{16}$: colorfulness of ROIs
	$f_{17}$: sharpness contrast between ROIs and background
	$f_{18}$: color contrast between ROIs and background
	$f_{19}$: brightness contrast between ROIs and background
	$f_{20}$: saturation contrast between ROIs and background
	$f_{21}=\max\left(f_{18},f_{19},f_{20}\right)$

**Table 7:** Overview of the proposed handcrafted features $F_{h}^{c}$ for CIAA.

Features	Formula
Global features	$f_{1}$: colorfulness
	$f_{2}$: min distance to intersection points (based on the
	rule of thirds) determined by sharpness values
	$f_{3}$: min distance to intersection points (based on the
	rule of thirds) determined by color saliency values
	$f_{4}$: min distance to intersection points (based on the
	rule of thirds) determined by brightness values
	$f_{5}=\min\left(f_{2},f_{3},f_{4}\right)$
	$f_{6}$: distribution of sharpness values
	$f_{7}$: distribution of color saliency values
ROI and background features	$f_{8}$: mean of gradient values of ROIs
	$f_{9}$: standard deviation of gradient values of ROIs
	$f_{10}$: mean of color saliency values of ROIs
	$f_{11}$: standard deviation of color saliency values of ROIs
	$f_{12}$: mean of saturation values of ROIs
	$f_{13}$: standard deviation of saturation values of ROIs
	$f_{14}$: mean of brightness values of ROIs
	$f_{15}$: standard deviation of brightness values of ROIs
	$f_{16}$: colorfulness of ROIs
	$f_{17}$: mean of gradient values of background
	$f_{18}$: colorfulness of background
	$f_{19}$: sharpness contrast between ROIs and background
	$f_{20}$: color contrast between ROIs and background
	$f_{21}$: brightness contrast between ROIs and background
	$f_{22}$: saturation contrast between ROIs and background
	$f_{23}=\max\left(f_{21},f_{22},f_{23}\right)$

**Table 8:** Overview of evaluation criteria for IAA. z=1.96 for 95% confidence interval and the number of samples *N* is 800, 400 and 400 for GIAA, LIAA and CIAA respectively. TP, FP, TN, FN are a number of images.

Evaluation Criteria	Formula
Accuracy	$A=\frac{TP+TN}{TP+FP+TN+FN}$
Confidence interval	$I_{a}=z\times \sqrt{\frac{(1-A)\times A}{N}}$
Lower accuracy	$A_{l}=A-I_{a}$
Upper accuracy	$A_{u}=A+I_{a}$

**Table 9:** Evaluations of IAA with and without image classification using handcrafted and learned features.

Feature Vector	*A*	$I_{a}$	$A_{l}$	$A_{u}$
**GIAA—IAA without image classification**				
$F_{h}^{a}$	0.785	0.028	0.757	0.813
$F_{h}^{a_{1}}$	0.845	0.025	0.820	0.870
$F_{h}^{a_{2}}$	0.800	0.028	0.772	0.828
$F_{l}^{a}$	0.921	0.018	0.903	0.939
**LIAA—IAA for large field images only**				
$F_{h}^{l}$	0.913	0.028	0.885	0.941
$F_{h}^{a_{1}}$	0.880	0.031	0.849	0.911
$F_{h}^{a_{2}}$	0.878	0.032	0.846	0.910
$F_{l}^{l}$	0.940	0.023	0.917	0.963
**CIAA—IAA for close-up images only**				
$F_{h}^{c}$	0.843	0.036	0.807	0.879
$F_{h}^{a_{1}}$	0.860	0.034	0.816	0.894
$F_{h}^{a_{2}}$	0.833	0.037	0.796	0.870
$F_{l}^{c}$	0.925	0.026	0.899	0.951

**Table 10:** The number of global features, RB features in the two feature sets $F_{l}^{l}$ and $F_{l}^{c}$ for LIAA and CIAA respectively.

Feature Set	The Number of	
	**Global Features**	**RB Features**
$F_{l}^{l}$	21	0
$F_{l}^{c}$	18	5

**Table 11:** Evaluations of CIAA using global features, RB features and both global features and RB features.

Feature Vector	*A*	$I_{a}$	$A_{l}$	$A_{u}$
**CIAA—IAA for close-up images only**				
$F_{l}^{c}$	0.925	0.026	0.899	0.951
$F_{g}^{c}$	0.908	0.028	0.880	0.936
$F_{rb}^{c}$	0.868	0.033	0.835	0.901

**Table 12:** Evaluations of LIAA, CIAA using Suran’s and Aydin’s global features, RB features and both global features and RB features.

Feature Vector	*A*	$I_{a}$	$A_{l}$	$A_{u}$
**LIAA using Suran’s features**				
$F_{h}^{a_{1}}$	0.880	0.031	0.849	0.911
$F_{g}^{a_{1}}$	0.875	0.032	0.843	0.907
$F_{rb}^{a_{1}}$	0.848	0.035	0.813	0.883
**CIAA using Suran’s features**				
$F_{h}^{a_{1}}$	0.860	0.034	0.826	0.894
$F_{g}^{a_{1}}$	0.853	0.035	0.818	0.888
$F_{rb}^{a_{1}}$	0.728	0.044	0.684	0.772
**LIAA using Aydin’s features**				
$F_{h}^{a_{2}}$	0.878	0.032	0.846	0.910
$F_{g}^{a_{2}}$	0.888	0.031	0.857	0.919
$F_{rb}^{a_{2}}$	0.540	0.049	0.491	0.589
**CIAA using Aydin’s features**				
$F_{h}^{a_{2}}$	0.833	0.037	0.796	0.870
$F_{g}^{a_{2}}$	0.740	0.043	0.697	0.783
$F_{rb}^{a_{2}}$	0.818	0.038	0.780	0.856

## Abbreviations

The following abbreviations are used in this manuscript:

CIAA	Close-up Image Aesthetic Assessment
CNN	Convolutional Neural Network
DOF	Depth Of Field
GIAA	General Image Aesthetic Assessment
IAA	Image Aesthetic Assessment
LCIC	Large field/Close-up Image Classification
LIAA	Large field Image Aesthetic Assessment
RB	Region of interest and Background
ROI	Region Of Interest
ROIE	Region Of Interest Extraction
SVM	Support Vector Machine
